# How Does Physical and Psychological Recovery Vary Among Competitive and Recreational Athletes After Anterior Cruciate Ligament Reconstruction?

**DOI:** 10.1177/19417381241249413

**Published:** 2024-05-12

**Authors:** Mandeep Kaur, Terese L. Chmielewski, Susan Saliba, Joe Hart

**Affiliations:** † School of Health Professions, Department of Physical Therapy and Rehabilitation Sciences, University of Texas Medical Branch, Galveston, Texas; ‡ TRIA Orthopedic Center, Bloomington, Minnesota; § Department of Kinesiology, University of Virginia, Charlottesville, Virginia; ‖ Department of Orthopaedic Surgery, University of North Carolina, Chapel Hill, North Carolina

**Keywords:** athletes, knee health, knee injury osteoarthritis outcome scale, strength

## Abstract

**Background:**

The recovery and rehabilitation journey after anterior cruciate ligament reconstruction (ACLR) surgery can be different for competitive and recreational athletes as their motivation and goals toward sports are different.

**Hypothesis:**

Competitive athletes would present with better patient-reported outcomes and higher muscle strength compared with recreational athletes postsurgery. Second, competitive athletes would recover better (patient-reported outcome [PRO] measures and muscle strength) compared with recreational athletes at later stages.

**Study Design:**

Cross-sectional laboratory-based study.

**Level of Evidence:**

Level 2.

**Methods:**

A total of 245 patients with unilateral ACLR were categorized as competitive or recreational athletes and grouped into early (4-6.9 months) or late (7-10 months) stages of recovery. PRO were collected for psychological response (Tampa Scale Kinesiophobia; Anterior Cruciate Ligament-Return to Sport after Injury), perceived knee function (International Knee Documentation Committee subjective form [IKDC]), and quality of life (Knee injury and Osteoarthritis Outcome Score; Veteran Rand-12). Isokinetic, concentric knee extension strength was measured bilaterally with a multimodal dynamometer (System 4, Biodex Medical Systems) at a speed of 90° and 180°/s.

**Results:**

Competitive athletes had significantly higher scores for IKDC (*P* = 0.03), and quadriceps peak torque at 90°/s (*P* = 0.01) and 180°/s (*P* < 0.01) compared with recreational athletes. Competitive athletes had higher quadriceps strength at 90°/s (*P* < 0.01) and 180°/s (*P* = 0.02) in the late group. Recreational athletes displayed higher sports participation in the late group.

**Conclusion:**

Outcomes of ACLR may differ based on preinjury athletic level. Whereas competitive athletes had higher knee and muscle function than recreational athletes, psychological measures were not different among groups.

**Clinical Relevance:**

There is a need for more individualized care for patients with ACLR since there is variability among patient goals postsurgery. This information might help set realistic expectations for competitive and recreational athletes after surgery.

Return to preinjury levels of physical activity or sports is one of the goals after anterior cruciate ligament (ACL) reconstruction (ACLR). Although 81% of patients return to any form of sports, only 55% of patients return to competitive participation after ACLR.^3^ Differences in rates of return to sports have been reported among elite athletes and nonathletes,^3–5^ such that elite athletes are twice as likely to return to their preinjury level of sports, and have 6 times the odds of returning to competitive sports compared with nonelite athletes.^1^ Level of sports participation is assessed by the Tegner Physical activity level scale, where higher scores indicate a high level of sports participation, and returning to the preinjury level of sports is the primary goal for those who undergo surgery. A recent study has indicated that only 24% of recreational athletes returned to their preinjury Tegner level 1 year post-ACLR.^20^

Recovery after ACLR can be different for competitive and recreational athletes as it involves an interplay among various physical and psychological factors.^31^ Psychological factors such as fear of reinjury, motivation to return to sports,^28^ higher levels of self-esteem,^11^ and psychological readiness can influence the overall outcome after surgery. In a study where 86% of participants stated their goal was to return to their preinjury sports, those who had returned to their preinjury sports activity at 52 weeks were more motivated and more satisfied with their activity.^28^ Competitive athletes may have higher goals in sports,^28^ along with greater motivation to perform well and anxiety supporting performance.^27^ However, this is not the desire for recreational athletes as motivation is dependent on the level of sports activity.^27^ Recreational athletes tend to be focused more on returning to their lives, including their jobs, but still intend to be involved in their day-to-day recreational activities and maintaining their quality of life. Understanding the differences in recovery and rehabilitation among competitive and recreational athletes might help clinicians set realistic expectations after surgery for their patients. The primary focus of current ACLR rehabilitation protocols is on achieving quadriceps strength symmetry^6^; indeed, only a few rehabilitation specialists use primarily patient-reported outcome (PRO) measures, with very few focused on measuring fear or athletic confidence scales.^16^ Considering the variation among rehabilitation practices, patient goals, psychological factors,^8,17^ subjective feelings of knee function,^21^ and other life commitments post-ACLR,^16^ the return-to-sports outcomes may differ overall.^2,14^ However, very few studies have explored how these constructs vary among different levels of athletes.

To improve and set realistic rehabilitation outcomes among competitive and recreational athletes postsurgery, PROs that document self-perceived knee function, psychological constructs, and muscle strength need to be explored. Screening for potentially modifiable contextual factors early after ACLR may help clinicians identify athletes who could be at risk of not returning to the preinjury level of sports. Clinicians can help athletes by developing a more individualized rehabilitation plan, leading to improved outcomes overall.^1^ Rehabilitation teams need to address physical and psychological factors when treating injured athletes if complete, holistic recovery is to occur. Therefore, the purposes of this study were to (1) compare PRO measures and quadriceps muscle strength in participants with ACLR in competitive and recreational athletes and (2) examine how these PROs and strength measures differ among participants who are at early and late stages of rehabilitation among competitive and recreational groups. It was hypothesized that competitive athletes would present with better PROs and higher muscle strength compared with recreational athletes postsurgery. Second, we hypothesized that competitive athletes would present with better recovery (PRO measures and muscle strength) at later stages of recovery compared with recreational athletes.

## Methods

This study was derived from a clinical data set of patients with ACLR in an academic institution from 2013 to 2019. These patients were referred for postoperative assessments consisting of muscle strength testing, functional performance tests, and PROs. Measures of quadriceps strength and PROs related to psychological response, perceived knee function, and health-related quality of life were included in this study.

### Participants

A total of 245 patients with primary isolated ACLR took part in the study. Inclusion criterion was men and women with unilateral ACLR between the ages of 16 and 40 years, treated with any type of graft. Patients were excluded from the study if they had lower extremity joint surgery before ACLR, a concomitant ligament reconstruction, a graft failure, or surgical complications from ACLR. Patients with meniscal repair or meniscectomy at the time of ACLR were not excluded from participation because of the high occurrence of these concomitant procedures.^9^ Although participants received rehabilitation at different health facilities, they each reported that they followed the protocol prescribed by their surgeons. The institutional review board approved this study and all patients provided written informed consent. Parents/guardians provided consent for those who were minors at the time of data collection.

#### Demographic Information

Demographic variables included age, height, mass, preinjury Tegner Activity Rating score, and time from surgery to testing. The Tegner Activity Rating Scale was used to provide a standardized method for determining the activity level before injury and the level of activity postinjury on a numerical scale. Patients with a Tegner score of ≥8 are classified as competitive athletes, and those with Tegner scores <8 are classified as recreational athletes.^12^ These criteria were used in the present study. Patients were also divided into the early stages of rehabilitation (4-6.9 months postsurgery) and later stages of rehabilitation (7-10 months postsurgery) groups based on the typical discharge from rehabilitation at around 6 or 7 months postsurgery.^10^

##### Patient-Reported Outcomes

All patients completed PROs related to psychological response, perceived knee function, and health-related quality of life.

The 17-item version of the Tampa Scale for Kinesiophobia (TSK-17) was used to measure kinesiophobia. TSK-17 is a reliable scale to assess kinesiophobia in patients with ACL injuries,^18^ and measures a patient’s psychological response to pain, or the anticipation of pain, that leads to an avoidance of movement due to fear of recurrent pain or injury.^32^ Each item is scored from 1 to 4 points for a total score that ranges from 17 to 68 points. Higher scores represent higher kinesiophobia.^32^

The Anterior Cruciate Ligament - Return to Sport after Injury (ACL-RSI) scale is a reliable and valid 12-item questionnaire to assess psychological readiness for sports participation in the domains of emotion, confidence in performance, and risk appraisal.^33^ The scale ranges from 1 to 10, with higher scores indicating a more positive psychological response. The total score was determined by adding the values of the 12 responses and then calculating their relationship to 100 to obtain a percentage.

The International Knee Documentation Committee subjective form (IKDC) is used to measure regional knee health in terms of knee function, and is reliable and valid for knee ligamentous injuries. Scores range from 0 to 100, where 100 indicates that a patient has no limitation with daily or sporting activities and an absence of symptoms.^19^ The IKDC demonstrates good internal consistency and test-retest reliability in patients with ACLR.^15^ The minimum clinically important difference (MCID) for the IKDC is 9 points.^22^

The Knee Injury and Osteoarthritis Score (KOOS) measures patients’ opinions about their knee and associated problems such as ligamentous injuries.^12,24^ It has 5 domains: (1) pain, (2) symptoms, (3) function during activities of daily living, (4) sport and recreational function, and (5) knee-related quality of life. Scoring ranges from 0% to 100%, where higher values indicate better knee function. KOOS subscales have good reliability and validity in patients with knee injuries. The MCID for the KOOS subscales is 8 points.^24^

Global health was measured using the Veterans Rand 12-Item Health Survey (VR-12) total score, which is a summed composite of mental and physical components.^26^ This questionnaire is responsive to knee conditions.^23^ Scores range from 0% to 100%, with higher values indicating better global health.

### Quadriceps Strength Testing

Isokinetic, concentric knee extension strength was measured bilaterally with a multimodal dynamometer (System 4, Biodex Medical Systems) at a speed of 90° and 180°/s. Participants completed practice trials on each limb for familiarization until they were confident. The uninjured side was tested first. The participants provided maximal effort through their full range of motion for 8 trials. Measures of mean peak torque for knee extension were expressed normalized to body mass (N·m/kg). The quadriceps limb symmetry index (LSI) was calculated using the following equation: (involved side peak torque/uninvolved side peak torque) × 100.

### Statistical Analysis

Dependent variables consisted of quadriceps peak torque and PRO measures such as IKDC, KOOS, Tegner Physical activity level scales, ACL-RSI, and TSK-17. The normality of the data was tested with histograms and Normal Q-Q plots. Data were found to be distributed normally with histograms and datapoints were close to the diagonal line in Q-Q plots. We report group means and standard deviations for normally distributed continuous variables. To explore the differences in recovery among competitive and recreational athletes after ACLR, an independent-samples *t* test was used. To explore whether PRO measures and muscle strength recovery differ among competitive and recreational athletes, a separate independent *t* test was used among both groups. To explore the differences in physical activity levels among competitive and recreational athletes before ACL injury and postsurgery, a nonparametric test (Mann-Whitney test) was used.

Differences between groups were reported as mean differences and 95% CIs. All statistical analyses were performed using SPSS statistical analysis software (IBM SPSS Statistics Version 28.0; IBM Corp), with *P* < 0.05 considered to be significant.

## Results

The sample consisted of 193 competitive and 52 recreational athletes. Descriptive data are presented in Table 1. Competitive athletes had significantly higher physical activity levels than recreational athletes at 6.3 months postsurgery (*P* < 0.01).

**Table 1.** Age, height, mass, and time since surgery for competitive and recreational athletes

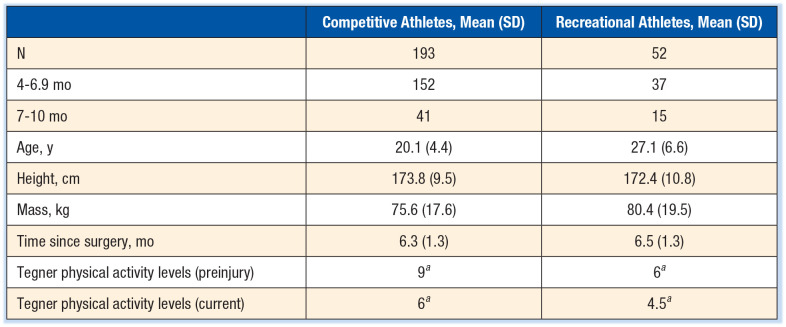

a Mann-Whitney test *P* < 0.01.

Competitive athletes had higher IKDC (*P* = 0.03) and ACL-RSI (*P* = 0.05) scores (Table 2) than recreational athletes. Competitive athletes had significantly higher peak torque quadriceps at 90°/s (*P* = 0.02) and at 180°/s (*P* < 0.01) than recreational athletes. No significant differences were found for any other PROs.

**Table 2.** PROs and muscle strength among competitive and recreational athletes after ACLR

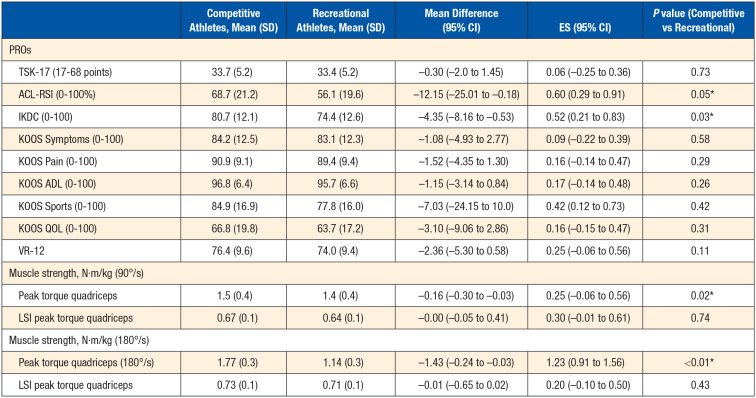

ACLR, anterior cruciate ligament reconstruction; ACL-RSI, Anterior Cruciate Ligament-Return To Sports Index; ES, effect size; IKDC, International Knee Documentation Committee; KOOS, Knee Injury and Osteoarthritis Outcome; LSI, limb symmetry index; PRO, patient-reported outcome; QOL, quality of life; VR, Veteran Rand.

* *P* ≤ 0.05 (mean difference is significant at the 0.05 level).

Competitive athletes were 5.8 months postsurgery on an average in the early group, and those at later stage were at 8.1 months postsurgery. No significant differences were found for any PROs between the early stages versus the later stages of rehabilitation groups (Table 3). Competitive athletes had significant differences in peak torque at 90°/sec (*P* < 0.01) and 180°/sec (*P* = 0.02) between the early and late group such that patients in late groups had higher peak torque.

**Table 3.** PROs and muscle strength among competitive and recreational athletes during early and late groups after ACLR

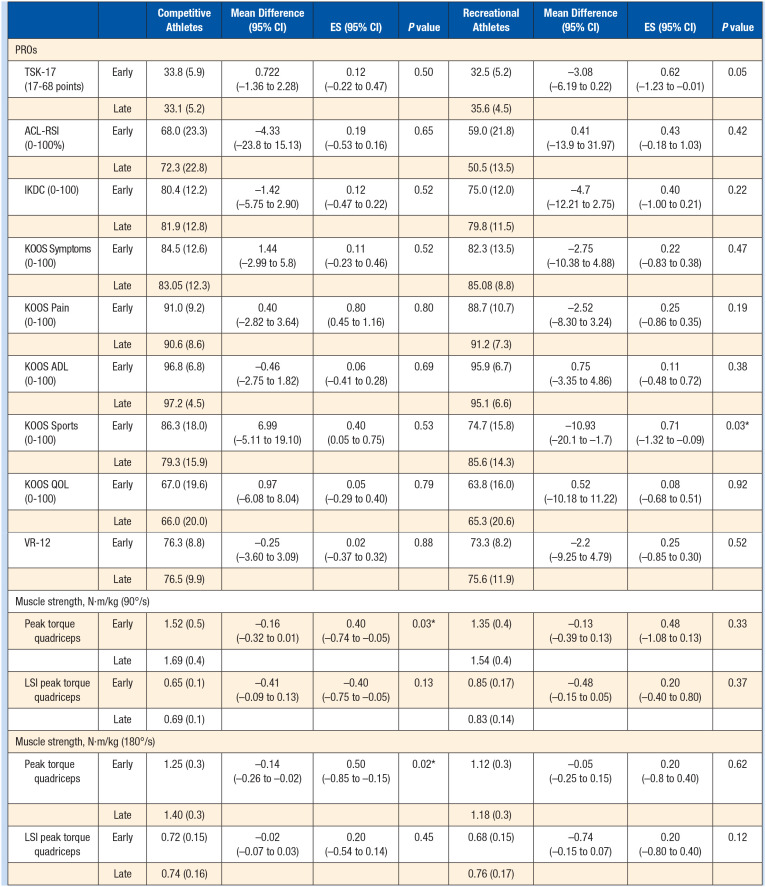

ACLR, anterior cruciate ligament reconstruction; ACL-RSI, Anterior Cruciate Ligament-Return To Sports Index; ADL, activities of daily living; ES, effect size; IKDC, International Knee Documentation Committee; KOOS, Knee Injury and Osteoarthritis Outcome; LSI, limb symmetry index; PRO, patient-reported outcome; QOL, quality of life; VR, veteran rand.

* *P* ≤ 0.05 (mean difference is significant at the 0.05 level).

Recreational athletes were 5.9 months postsurgery on average in the early group, and those in the late group were 7.9 months postsurgery. Recreational athletes had significant differences for the KOOS Sports subscales (*P* = 0.03) (Table 3) such that patients in the late group had higher scores.

## Discussion

One of the goals of this study was to compare PROs and quadriceps strength in competitive and recreational athletes after ACLR. Competitive athletes had better knee function recovery as indicated by IKDC scores and higher peak torque quadriceps (90 and 180°/s) compared with recreational athletes. Competitive athletes are generally highly motivated to return to sports and this may prompt them to undergo intensive rehabilitation, which might have led to better strength gains in this group. It should be noted that magnitude of strength differences between competitive and recreational athletes were small (1.5 vs 1.4 N·m/kg) and the potential clinical impact of these differences on long-term outcomes after ACLR should be the focus of further research. There is a paucity of research focused on exploring the outcomes of ACLR postsurgery among different levels of athletes, for instance, competitive versus recreational athletes. However, a study that explored strength gains at 19 months postsurgery reported higher strength among competitive athletes compared with recreational athletes.^7^ Further research is warranted to explore whether mid- to long-term outcomes vary among these groups, especially with the new surgical techniques and more rigorous rehabilitation being followed in the past few years.

This study also sought to explore differences in recovery in PROs and muscle strength among competitive and recreational athletes for those who are at early and later stages of rehabilitation groups to understand whether overall outcomes are different by the end of recovery. Among the competitive athletes, peak torque quadriceps was higher in the late group. The findings should be interpreted with caution as the early and late groups in this study represent the different athletes at two different stages of rehabilitation. However, better quadriceps strength outcomes as seen in the late group among competitive athletes can be associated with improved knee function and movement patterns helping them to return to sports.^25^ Similarly, the LSI was higher for the recreational athletes; again, this needs to be interpreted with caution as LSI is a ratio and depends on the peak torque on the uninjured side too. Among the participants in the recreational group, those in the late group indicated higher sports function as per the KOOS Sports subscale. This might be due to the lower levels of sports participation needed to return for recreational purposes. This finding should be understood while considering that the physical activity levels of recreational athletes were lower than those of competitive athletes as per Tegner physical activity level scores (competitive athletes, 6; recreational, 4.5). Apart from the KOOS Sports scale, no significant differences were found for any other PRO variables from the early to later stages of rehabilitation. This finding may be due to the grouping used in this study, where the early group of rehabilitation was up to 6.9 months postsurgery, and most of the rehabilitation progression had already occurred for the outcomes being tracked.

It should be noted that one of the goals for athletes who undergo ACLR is to return to sports; PROs with scores such as IKDC ≥ 85, ACL-RSI ≥ 65, and LSI for quadriceps scores ≥90 are considered as one of the indicators of satisfactory recovery to achieve before releasing to sports.^13^ This study found that competitive athletes were closer to these goals at 6 months postsurgery. This information might be helpful to the rehabilitation team for goal setting among different levels of athletes.

TSK did not differ significantly from early to later stages for any of the groups in our study. A plausible explanation is that participants of our study were 6.5 months postsurgery and, by this time during rehabilitation, participants are usually not practicing their sports. Athletes are more likely to experience greater kinesiophobia while encountering difficult situations for the knee such as twists or turns on the injured side. Also, our study found no significant differences in kinesiophobia among competitive and recreational athletes postsurgery. Participants had a wide range of TSK scores (20-50 points), ranging from those with no kinesiophobia to high levels of kinesiophobia scores. This variation in scores highlights the need to monitor kinesiophobia through serial assessments by the healthcare team during rehabilitation and potentially address it with alternative intervention methods. The influence of kinesiophobia on return to sports is already known, and it is thought to affect the gait pattern as well.^30^ From the results of this study, kinesiophobia needs to be addressed since it does not resolve itself.

This study provides insight regarding the recovery of quadriceps muscle strength and other PROs related to knee function and psychological readiness among competitive and recreational athletes after ACLR. This information may help clinicians in setting realistic goals after ACLR. In addition, this study found that there were no differences in kinesiophobia scores based on the groups (competitive or recreational). Clinicians need to actively screen their patients to identify who will need more help to overcome fear during rehabilitation. Anxiety screening should be done sporadically during rehabilitation.

### Strengths and Limitations

This study has significant limitations. Patients with all types of grafts were included in the trial, and graft site weakness affects the quadriceps muscle strength (bone-patellar tendon-bone autograft).^29^ We used VR-12 total score, rather than separate scores of mental and physical components, which limits the interpretation. Separate physical and mental components may provide a better understanding of global knee health.

## Conclusion

Competitive athletes had better knee function and higher quadriceps strength compared with recreational athletes. Among competitive athletes, those in the later stages of the rehabilitation had higher peak torque than those during the early stages of the rehabilitation. Recreational athletes had more sports participation in the later stages of rehabilitation. No differences were found in kinesiophobia among both groups. Therefore, clinicians need to identify patients with these issues during rehabilitation so that proper interventions can be applied to set realistic expectations for competitive and recreational athletes.
